# Trisomy 18 with widespread calcinosis cutis

**DOI:** 10.1093/skinhd/vzaf023

**Published:** 2025-04-22

**Authors:** Airin Sato, Yu Matsui, Teruhiko Makino, Tadamichi Shimizu

**Affiliations:** Department of Dermatology, Faculty of Medicine, Academic Assembly, University of Toyama, Toyama, Japan; Department of Dermatology, Faculty of Medicine, Academic Assembly, University of Toyama, Toyama, Japan; Department of Dermatology, Faculty of Medicine, Academic Assembly, University of Toyama, Toyama, Japan; Department of Dermatology, Faculty of Medicine, Academic Assembly, University of Toyama, Toyama, Japan

## Abstract

Trisomy 18 is the second most common autosomal trisomy, associated with high mortality, with only 5–10% of affected individuals surviving beyond the first year of life. Consequently, comorbidities in long-term survivors are rarely reported. We describe the case of a 6-year-old East Asian girl with trisomy 18 who presented with calcinosis cutis. The patient initially developed a firm, non-tender subcutaneous nodule on the left wrist, which later spread to the forearm and upper arm. Physical examination and imaging revealed extensive subcutaneous nodules in other extremities. High-frequency ultrasonography showed hypoechogenic masses with posterior acoustic shadows, while skin biopsy revealed fat necrosis and calcium deposition. No significant abnormalities were detected in the levels of calcium, phosphorus, parathyroid hormone or vitamin D. Based on laboratory findings and the patient’s medical history, metabolic, nutritional, inflammatory and iatrogenic causes of calcinosis cutis were unlikely. Consequently, the condition was classified as either dystrophic or idiopathic calcinosis cutis. The patient was managed as an outpatient without specific treatment. This report discusses the mechanisms of calcinosis cutis and the reasons for the limited research on its association with trisomy 18. To our knowledge, this is the first report describing calcinosis cutis as a concomitant condition in a paediatric patient with trisomy 18, and it is anticipated that increased awareness of this disease may lead to a rise in reported cases in the future.

## Teaser

Rapid advancements in neonatal care have resulted in a long-term survival in cases of trisomy 18, and few reports of dermatological comorbidities exist. Organ calcification during the fetal stage is known to be associated with chromosomal abnormalities. We describe the case of a 6-year-old East Asian girl with trisomy 18 who presented with calcinosis cutis.

## Case report

Trisomy 18 is the second most common autosomal trisomy and is associated with a high mortality rate, with only approximately 5–10% of affected individuals surviving beyond the first year of life. Hence, there are few reports on comorbidities in long-term survivors.^1^ We describe the case of a 6-year-old East Asian girl with trisomy 18, who presented with calcinosis cutis.

**Figure 1:**
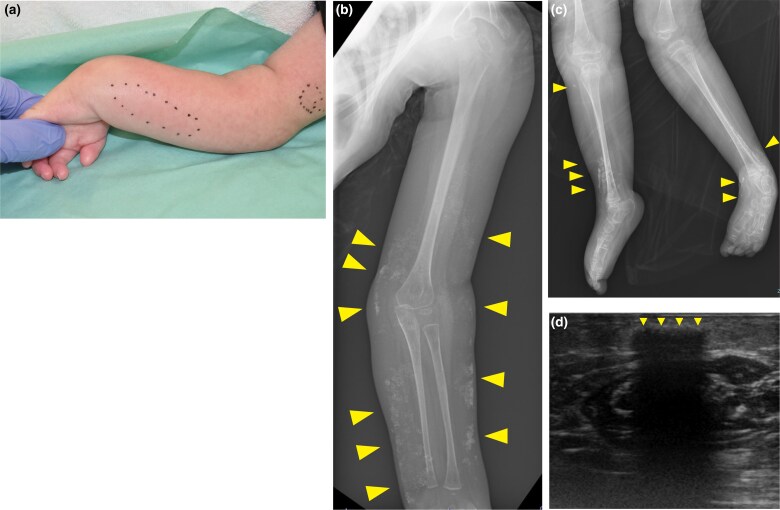
(a) Nontender subcutaneous nodules in the left arm. No significant changes in the epidermis were observed. (b, c) Radiography revealed extensive subcutaneous nodules (arrowhead) in the extremities. (b) Left arm. (c) Lower limb. (d) High-frequency ultrasonography showed hypoechogenic masses with posterior acoustic shadows (arrowhead).

**Figure 2:**
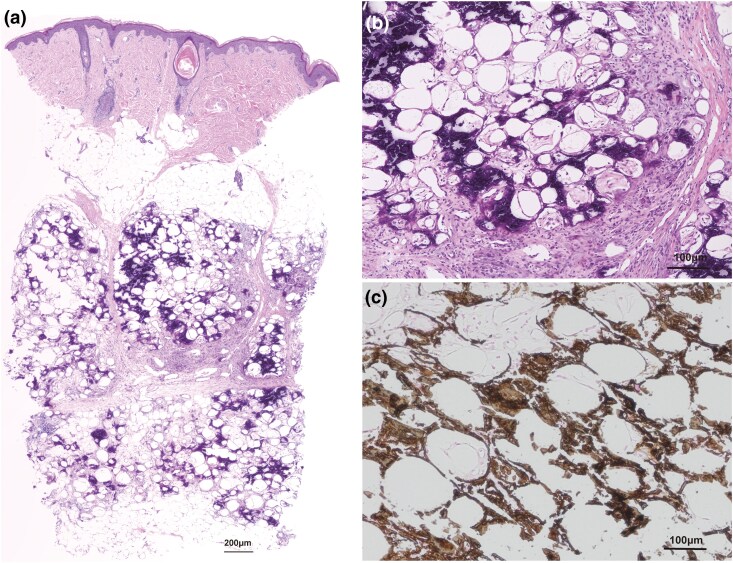
(a, b) Histopathological examination of the indurated area of the left forearm. Basophilic changes were observed in subcutaneous adipose tissue (haematoxylin–eosin staining, scale marker: 200 μm, 100 μm) (c) von Kossa staining revealed that the basophilic deposits were due to calcium salts (scale marker: 100 μm).

A firm, nontender subcutaneous nodule was observed on the patient’s left wrist 1 month previously. Thereafter, nodules developed on the forearm and upper arm (Figure 1a). Although the patient’s primary complaint was localized to the left upper limb, a physical examination and radiography revealed extensive subcutaneous nodules in the other extremities (Figure 1b, c). High-frequency ultrasonography showed hypoechogenic masses with posterior acoustic shadows and an absence of Doppler signals (Figure 1d). Skin biopsy showed extensive fat necrosis with mild inflammatory cell infiltration, primarily lymphocytes, around adipocytes and basophilic changes within the subcutaneous fat lobules, suggesting subcutaneous calcification without thrombi (Figure 2a, b). Kossa staining also revealed that this was due to the presence of calcium salts (Figure 2c). The patient had no relevant family history. Her medical history included trisomy 18 complications, including being small for her gestational age, epilepsy and heart defects (i.e. ventricular and atrial septal defects). No congenital morphological abnormalities were detected during routine neonatal screening at the time of birth. There were no changes in her medications, including antiepileptics, and no history of trauma, muscle weakness, rash, joint pain or dysphagia. Blood test results for calcium (8.5 mg/dL; normal range: 8.8–10.1) and phosphorus (4.7 mg/dL; normal range: 2.7–4.6), as well as alkaline phosphatase, creatinine, urea nitrogen, vitamin D and parathyroid hormone, were within the normal limits. Tests for antinuclear antibodies yielded negative results. Based on these findings, metastatic and iatrogenic calcinosis cutis were ruled out. Generally, dystrophic calcinosis cutis occurs secondary to autoimmune diseases, malignancies such as basal cell carcinoma, infections or trauma. Although there was no history of trauma or findings suggestive of an autoimmune disorder in this case, extensive fat necrosis was observed on histopathology, making it difficult to completely exclude this possibility. Therefore, the condition was considered to be either dystrophic or idiopathic calcinosis cutis. The patient was followed up as an outpatient without treatment.

Previously, treatment for trisomy 18 was categorized as ‘any life-prolonging medical interventions should be restricted upon the diagnosis’ due to the severe prognosis and significant developmental delays.^2^ Subsequently, rapid advancements in neonatal care and the management of congenital heart defects have led to a re-evaluation of the efficacy of medical interventions for trisomy 18, resulting in cases of long-term survival.^3,4^ However, there are few clinical reports on associated comorbidities due to its rarity and the limited number of long-term survivors.

Currently, two primary mechanisms have been proposed for the pathogenesis of calcinosis cutis. In mechanism 1, changes in cellular activity and structural alterations in tissues contribute to calcified lesions. Subsequent reabsorption leaves calcified granules that – along with tissue changes – form calcified nodules.^5^ In mechanism 2, calcification of pre-existing skin structures (e.g. milia, syringoma and naevus) occurs.^6^

In the present case, Mechanism 1 is more likely, as no subcutaneous calcification of the extremities was observed at birth. Although chromosomal abnormalities have been linked to fetal tissue calcification, with organ calcification also documented in trisomy 18, the exact mechanisms remain unclear.^7^ Interestingly, trisomy 18 has been identified in a subset of pilomatricomas, pointing to a possible role of chromosomal abnormalities in abnormal cellular processes.^8^

In addition, milia-like idiopathic calcinosis cutis is a well-documented form of cutaneous calcification and is classified as idiopathic calcinosis cutis. It is frequently reported in individuals with trisomy 21, likely due to its higher birth prevalence and increased life expectancy.^9^ Despite these findings, studies have yet to clarify the pathophysiological mechanisms linking chromosomal abnormalities and cutaneous calcification.^7,10^

To extend the discussion beyond chromosomal abnormalities, inflammation is often implicated in the pathogenesis of ectopic calcification across various tissues, including the skin.^11^ Although serum calcium levels remain normal in most cases of ectopic calcification, this pattern has also been documented in other medical fields, particularly in orthopaedic research.^12^ Recognized contributing factors include alterations in local pH and oxygen levels, as well as the presence of mineralization nuclei, both of which are associated with inflammation and tissue injury.^12^ In the present case, extensive fat necrosis was observed. While fat necrosis is often associated with pancreatitis, trauma, circulatory disorders, infections, inflammatory diseases or drug use, these common causes were ruled out based on the patient’s age, medical history and lifestyle.^13^ Given the proposed mechanisms of ectopic calcification, the inflammatory response induced by fat necrosis may have played a role in promoting calcium deposition. However, much of the knowledge about ectopic calcifications derives from experimental studies on calcifications in the arterial media and heart valves, highlighting the complexity of its pathogenesis and suggesting that multiple factors contribute to the development of such lesions.

Although the biopsied site exhibited fat necrosis, calcinosis cutis was also observed in other extremities in this case. This widespread presentation suggests a systemic factor rather than a localized response. Considering the association between chromosomal abnormalities and fetal tissue calcification, including organ calcification in trisomy 18, it is plausible that genetic factors may also contribute to the development of cutaneous calcinosis in this case.^14^

While the possibility that calcinosis cutis is an incidental finding cannot be completely excluded, the exceedingly low prevalence rates of this chromosomal anomaly and paediatric cutaneous calcinosis (0.0001% and 0.026%, respectively) make such coincidence appear unlikely.^1,14^

There are several reasons for the lack of studies reporting an association between trisomy 18 and calcinosis cutis. (i) The low survival rate of trisomy 18 makes long-term observation difficult. Calcinosis cutis appears gradually, and patients die before it can be detected. (ii) In patients with trisomy 18, severe complications often occur in crucial organs such as the heart and brain, and the management of skin abnormalities tends to be a lower priority. Consequently, calcinosis cutis is rarely detected or recognized even if present at birth. (iii) The detection and recognition of calcinosis cutis are challenging, as it often progresses slowly and is not visible. (iv) Radiographic examinations in children are limited due to the risk of radiation exposure, despite the usefulness of radiography for detecting calcinosis cutis.

After diagnosis, various pharmacological and surgical treatment options have been proposed for managing calcinosis cutis, including calcium channel blockers, bisphosphonates, warfarin, intravenous immunoglobulin and anti-tumour necrosis factor agents. However, their efficacy remains limited, and no standardized treatment has been established. Surgical excision remains the most definitive approach but carries the risk of recurrence.^15^

While advances in medical care have improved the prognosis of trisomy 18 in recent years, many affected individuals still require extensive invasive treatments. Therefore, although more aggressive therapeutic interventions could be considered, a more practical approach may be for healthcare providers and families to share a thorough understanding of potential dermatological complications and proceed with careful long-term observation.

To our knowledge, this is the first report describing calcinosis cutis as a concomitant condition in a paediatric patient with trisomy 18, the incidence of which may increase in the future. The further accumulation of reports is needed to improve our understanding of the condition, including its pathophysiological mechanisms.

## Data Availability

No data were generated.
